# On GRAND-Assisted Vector Random Linear Network Coding in Wireless Broadcasts

**DOI:** 10.3390/e28040450

**Published:** 2026-04-15

**Authors:** Rina Su, Chengji Zhao, Qifu Sun, Linqi Song

**Affiliations:** 1 School of Computer and Communication Engineering, University of Science and Technology Beijing, Beijing 100083, China; rinasu@ustb.edu.cn (R.S.); m202421003@xs.ustb.edu.cn (C.Z.); 2 Department of Computer Science, City University of Hong Kong, Hong Kong; linqi.song@cityu.edu.hk; 3 City University of Hong Kong Shenzhen Research Institute, Shenzhen 518057, China

**Keywords:** random linear network coding, vector linear network coding, guessing random additive noise decoding, syndrome decoding, completion delay, wireless broadcasts

## Abstract

Recent works have combined random linear network coding (RLNC) with guessing random additive noise decoding (GRAND) to leverage RLNC packets to partially correct bit errors prior to RLNC decoding, so as to reduce the packet erasure rates in wireless broadcast networks. However, existing schemes are restricted to scalar RLNC over the finite field $\mathrm{GF}(2^{L})$. In this paper, we first formulate a general GRAND-assisted decoding framework for vector RLNC over the vector space $\mathrm{GF}{\left(2\right)}^{L}$, and further propose a design rule for vector RLNC schemes such that estimated error vectors can be efficiently obtained without incurring any additional computational overhead. Necessary and sufficient conditions for the correctness of every efficiently obtained estimated error vector are characterized. Two explicit vector RLNC schemes satisfying the proposed design rule are constructed. The first scheme is designed based on the matrix representation of GF($2^{L}$), and analytical results show that it achieves the same completion delay performance as the counterpart scalar RLNC scheme over GF($2^{L}$), while achieving up to a 37.3% reduction in coding computational complexity compared with the scalar one. The second scheme is designed based on sparse coding coefficient matrices. It further reduces computational complexity by up to 33.6% compared with the first scheme, at the cost of a slight degradation in completion delay performance.

## 1. Introduction

In wireless broadcast networks with packet erasures, maintaining timely information delivery is a key challenge. Metrics such as transmission delay have therefore attracted increasing attention. Network coding has emerged as an effective technique to address these challenges, as it allows multiple packets to be jointly encoded and transmitted, thereby reducing the number of transmissions. Scalar linear network coding (LNC) treats every data symbol, which is a binary sequence of length *L*, as an element in the finite field $\mathrm{GF}(2^{L})$, and performs linear combinations over $\mathrm{GF}(2^{L})$, with coding coefficients selected from the same field. Vector LNC [1,2] generalizes this framework by representing data symbols in the vector space $\mathrm{GF}{\left(2\right)}^{L}$ and applying coding coefficients in the form of L×L matrices over GF(2) to generate coded packets. While scalar LNC provides $2^{L}$ possible coding coefficients, vector LNC enlarges the set of available coefficients to $2^{L^{2}}$, offering substantially greater coding flexibility. The advantages of vector LNC over scalar LNC have been demonstrated in various wireline network settings. In particular, there exist networks whose capacity can be achieved by vector LNC but not by scalar LNC [1,3,4,5]. Moreover, for single-source multicast networks, explicit instances have been designed to demonstrate that the code dimension *L* required by vector LNC to achieve the multicast capacity can be much smaller compared with (that by) scalar LNC [6,7,8,9,10]. In addition, in the practical implementation of MDS codes over GF $\left(2^{m}\right)$ for data storage systems, several coding libraries have adopted the vector linear code versions based on matrix representation of $\mathrm{GF}\left(2^{m}\right)$, such as Jerasure Library [11] and Intel Intelligent Storage Acceleration Library [12] (ISA-L) for Reed–Solomon codes and Longhair library [13] for Cauchy Reed–Solomon codes.

Random LNC (RLNC) [14] is a widely studied LNC technique that can significantly improve the completion delay performance, a key metric of transmission efficiency in wireless networks with packet erasures [15,16,17,18,19,20,21,22]. Even though most attention has been focused on scalar RLNC, vector RLNC with properly designed coding coefficient sets has been shown to offer lower computational complexity [18,23]. In recent years, in the context of wireless broadcast networks, a series of works [24,25,26] investigated the joint design of RLNC with an interesting guessing–decoding principle, i.e., guessing random additive noise decoding (GRAND) [27,28,29], to exploit RLNC packets to partially correct certain bit errors in a packet before RLNC decoding, so that the packet erasure rate is reduced and thus completion delay performance is improved. However, these GRAND-assisted RLNC schemes only apply to scalar RLNC. In particular, the works in [24,25] investigated GRAND-assisted RLNC over GF(2), while [26] extended the study to $\mathrm{GF}(2^{L})$. To the best of our knowledge, no previous research has investigated GRAND-assisted vector RLNC schemes. This paper aims to bridge this gap. Compared with prior scalar GRAND-assisted RLNC schemes, the proposed vector RLNC framework enables comparable completion delay performance under appropriate design, while offering better design flexibility of coding coefficients so as to further reduce the coding complexity. The main contributions of this paper are enumerated as follows.

- We formulate a general framework of the GRAND-assisted decoding process of vector RLNC over GF${\left(2\right)}^{L}$ in wireless broadcasts.
- Building on this framework, we further establish a general design rule for vector RLNC schemes such that the estimated error vectors can be directly obtained without incurring any additional computational overhead. We further theoretically characterize necessary and sufficient conditions for the correctness of every efficiently obtained estimated error vector.
- Two explicit vector RLNC schemes satisfying the proposed design rule are deliberately constructed. The coding coefficient matrices in the first scheme are constructed by leveraging the matrix representation of GF($2^{L}$), while the second scheme employs sparse coding coefficient matrices.
- The first proposed scheme with the efficient GRAND process to obtain estimated error vectors is proved to have the same completion delay performance as the counterpart scalar RLNC scheme investigated in [26], while reducing coding computational complexity by up to 37.3%. Moreover, analytical and numerical results demonstrate that the second proposed vector RLNC scheme can further reduce computational complexity by up to 33.6% compared with the first one, at the cost of a slight degradation in completion delay performance.

The organization of this paper is as follows. Section 2 describes the network model, followed by Section 3 which establishes a general framework for GRAND-assisted vector RLNC. Section 4 proposes a design rule for the coding coefficient set of vector RLNC so that estimated error vectors can be efficiently obtained while avoiding any additional computational overhead. Explicit design examples subject to the rule are also presented. Section 5 numerically compares the two design examples. Section 6 concludes the paper.

## 2. Network Model

Consider a single-hop broadcast scenario with no feedback, where a single sender serves *R* receivers by transmitting *P* original packets. Every packet consists of *M* bits. Packet transmissions take place in discrete timeslots, with one packet broadcast in each slot. The sender-to-receiver links are independently modeled as memoryless packet erasure channels; in particular, the erasure probability for receiver *r* is $1-p_{r}$. The task of every receiver is to recover all *P* original packets successfully.

Following the settings in [15,16,18,19,21,22], this paper considers *systematic* RLNC transmission. In the first phase, the sender broadcasts the *P* original packets sequentially. After this systematic phase, coded transmissions begin, where each transmitted packet is generated as a random linear combination of the *P* original packets. The transmission proceeds until every receiver successfully recovers all *P* packets. To evaluate the throughput performance, we adopt *completion delay* as the primary metric, defined as the total number of coded packets transmitted by the sender.

We consider *vector RLNC* schemes defined over the *L*-dimensional vector space $\mathrm{GF}{\left(2\right)}^{L}$. Each packet $m_{j}$ containing *M* bits is represented as a row vector $\left[m_{j,1},m_{j,2},\ldots ,m_{j,M/L}\right]$, which consists of M/L symbols. Each symbol $m_{j,i}$ is viewed as an *L*-bit row vector over GF(2). Given an L×L matrix K over GF(2), the vector coding operation $K\circ m_{j}$ is defined symbol-wise as

(1) $$K\circ m_{j}=\left[m_{j,1}K,m_{j,2}K,\ldots ,m_{j,M/L}K\right].$$

For d≥1, the $d^{\mathrm{th}}$ coded packet $m_{P+d}$ randomly produced by the sender in the second phase can be represented as

(2) $$\begin{array}{cc} m_{P+d}= & \sum_{j=1}^{P}K_{d,j}\circ m_{j}\\ = & [\sum_{j=1}^{P}m_{j,1}K_{d,j},\ldots ,\sum_{j=1}^{P}m_{j,M/L}K_{d,j}]. \end{array}$$

where the coding coefficient matrices $K_{d,j}$ are randomly selected, according to certain defined rules, from L×L matrices over GF(2). The *global encoding kernel* of a packet is defined as a PL×L matrix over GF(2), which can also be interpreted as a P×1 block matrix where each block entry is an L×L coding coefficient matrix. For each original packet $m_{j}$ (1≤j≤P), its global encoding kernel $F_{j}$ is structured such that the $j^{\mathrm{th}}$ block entry is the identity matrix $I_{L}$, and all other block entries are L×L zero matrices. By construction, the concatenation of these kernels satisfies ${\left[F_{j}\right]}_{1\leq j\leq P}=I_{PL}$. For a coded packet $m_{P+d}=\sum_{j=1}^{P}K_{d,j}\circ m_{j}$, the corresponding global encoding kernel $F_{P+d}$ is formulated as $F_{P+d}={\left[K_{d,1}^{T}\;K_{d,2}^{T}\;\ldots \;K_{d,P}^{T}\right]}^{T}$. Denote by I the index set of packets successfully received by a receiver. In vector RLNC, the receiver can reconstruct all *P* original packets provided that the column-wise concatenation ${\left[F_{j}\right]}_{j\in I}$ of the |I| global encoding kernels forms a PL×|I|L matrix over GF(2) of rank PL.

## 3. A General Framework for GRAND-Assisted $\mathrm{GF}{\left(2\right)}^{L}$-RLNC Decoding

In most previous studies of RLNC in wireless broadcasts, only the successfully received packets will be utilized by a receiver. In these studies, receivers are assumed to discard the packets that are corrupted even for 1 bit, and to utilize only error-free packets for RLNC decoding. However, it has been advocated in [24,25,26] that both error-free and erroneous packets can be jointly utilized to generate syndromes so that some erroneous packets can be corrected following the principle of GRAND, and hence the actual packet erasure probability can be reduced. In [24,25,26], the GRAND-assisted decoding process is investigated in the context of scalar RLNC; that is, RLNC over GF(2) or GF($2^{L}$). In this section, we shall formulate, for the first time in the literature, the framework of GRAND-assisted RLNC decoding that applies to vector RLNC over GF${\left(2\right)}^{L}$.

To ease the ensuing presentation, unless otherwise specified, a fixed receiver *r* is assumed, so that *r* will not be reflected in notations. For every packet $m_{j}=\left[m_{j,1},\ldots ,m_{j,M/L}\right]$, let ${\hat{m}}_{j}=\left[{\hat{m}}_{j,1},\ldots ,{\hat{m}}_{j,M/L}\right]$ denote the packet received by receiver *r*, and let $e_{j}=\left[e_{j,1},\ldots ,e_{j,M/L}\right]$ denote the error vector ${\hat{m}}_{j}+m_{j}$, where every $e_{j,i}$ is called an error symbol. We have $\mathrm{Pr}\left\{e_{j}=0\right\}=\mathrm{Pr}\left\{m_{j}={\hat{m}}_{j}\right\}=p_{r}$. Following a similar assumption adopted in [24], we consider that upon receiving a packet ${\hat{m}}_{j}$, every receiver can categorize the packet as either error-free or erroneous by using an error detection mechanism such as cyclic redundancy check (CRC), and the global encoding kernel of the packet is identified at the receiver in an error-free manner.

When all *P* original packets are correctly received by receiver *r* then the transmission completes at *r*. Otherwise, let I⊆{1,…,P} denote the index set of the correctly received original packets and J={1,…,P}∖I denote the index set of the erroneously received original packets; that is, $I=\{1\leq j\leq P:m_{j}={\hat{m}}_{j}\}$, $J=\{1\leq j\leq P:m_{j}\neq {\hat{m}}_{j}\}$. Receiver *r* repeatedly performs the following routine every time a coded packet ${\hat{m}}_{P+d}$ is received, till all *P* original packets can be recovered; that is, $\mathrm{rank}({\left[F_{j}\right]}_{j\in I})=PL$.

- Upon receiving a new coded packet ${\hat{m}}_{P+d}=\sum_{j=1}^{P}K_{d,j}\circ m_{j}+e_{P+d}$, d≥1, if $m_{P+d}={\hat{m}}_{P+d}$, then receiver *r* updates the correct packet set I=I∪{P+d}; otherwise, receiver *r* updates the erroneous packet set J=J∪{P+d}.
- A *syndrome* $s_{d}=\left[s_{d,1},\ldots ,s_{d,M/L}\right]$ is computed as
  (3) $$\begin{array}{c} s_{d}=\sum_{j=1}^{P}K_{d,j}\circ {\hat{m}}_{j}+{\hat{m}}_{P+d}, \end{array}$$
  in which every symbol $s_{d,i}$, 1≤i≤M/L is equal to $\sum_{j=1}^{P}{\hat{m}}_{j,i}K_{d,j}+{\hat{m}}_{P+d,i}$. It can be readily checked that
  (4) $$\begin{array}{cc} s_{d}= & \sum_{j=1}^{P}K_{d,j}\circ e_{j}+e_{P+d},\\ s_{d,i}= & \sum_{j=1}^{P}e_{j,i}K_{d,j}+e_{P+d,i}. \end{array}$$
- Based on *d* syndromes $s_{1},\ldots ,s_{d}$, receiver *r* attempts to obtain an estimated error vector ${\tilde{e}}_{j}$ for every (nonzero) $e_{j}$, j∈J. According to the principle of GRAND,
  (5) $$\begin{array}{cc} \; & {\left[{\tilde{e}}_{j}\right]}_{j\in J}={\mathrm{argmax}}_{{\left[w_{j}\right]}_{j\in J}}\mathrm{Pr}\left\{e_{j}=w_{j},\forall j\in J\right\}\\ \; & \mathrm{subject}\;\mathrm{to}\; \end{array}$$
  (6) $$\begin{array}{cc} \; & w_{P+i}+\sum_{1\leq j\leq P,j\in J}K_{i,j}\circ w_{j}=s_{i}\;\forall 1\leq i\leq d \end{array}$$
  (7) $$\begin{array}{cc} \; & w_{j}\neq 0\;\forall j\in J,\;w_{P+i}=0\;\forall P+i\notin J \end{array}$$
- For each j∈J, check whether ${\tilde{e}}_{j}+{\hat{m}}_{j}$ is equal to $m_{j}$ by another round of CRC. If so, then receiver *r* successfully corrects $e_{j}$ and recovers packet $m_{j}$, removes *j* from J, puts *j* into I, sets $e_{j}=0$, and performs the following steps. If *j* is no larger than *P*, then update every syndrome $s_{i}$, 1≤i≤d as $s_{i}+K_{i,j}\circ {\tilde{e}}_{j}$; otherwise, update the syndrome $s_{j-P}$ as $s_{j-P}+{\tilde{e}}_{j}$.
- Based on the |I|=P+d−|J| correctly obtained packets, full recovery of all *P* original packets is possible when $\mathrm{rank}({\left[F_{j}\right]}_{j\in I})=PL$. Otherwise, receiver *r* will receive a new coded packet ${\hat{m}}_{P+d+1}$, with the above routine being repeated.

In the above formulation, estimated error vectors ${\tilde{e}}_{j}$, j∈J, are obtained based on the probability $\mathrm{Pr}\{e_{j}=w_{j}\}$ in (5), which is related to the underlying channel modeling for bit-level transmission. In previous studies of GRAND-assisted RLNC, memoryless binary symmetric channels (BSCs) were considered in [25,26], and Gilbert–Elliott channels were considered in [24]. Different channel models lead to different relationships between packet erasure rate $1-p_{r}$ and bit error rate in a packet, and thus lead to different $\mathrm{Pr}\{{\tilde{e}}_{j}=e_{j}\}$.

## 4. Efficient GRAND-Assisted Vector RLNC

As indicated by the formulation in (5)–(7) in Section 3, the computational complexity to obtain estimated error vectors ${\tilde{e}}_{j}$, j∈J, is excessively high. In the context of scalar RLNC, a novel scheme over GF($2^{L}$) was proposed in [26] so that estimated error vectors could be guessed with negligible computational overhead from syndromes. Motivated by this scalar scheme, in this section, we shall first introduce a general rule to design vector RLNC schemes over GF${\left(2\right)}^{L}$ for L>1, so that estimated error vectors can be obtained from syndromes without introducing any computational overhead. Subsequently, in Section 4.2 and Section 4.3, we explicitly design two vector RLNC schemes satisfying the general rule. The two concrete design instances exhibit different complexity and performance characteristics.

### 4.1. Design Rule of Vector RLNC and Efficient Estimate of ${\tilde{e}}_{j}$

In an error vector $e_{j}=\left[e_{j,1},\ldots ,e_{j,S}\right]$ consisting of *S* error symbols, if an error symbol $e_{j,i}$ contains exactly 1-bit error, it means that $e_{j,i}\in \mathrm{GF}{\left(2\right)}^{L}$ represents a unit vector of size *L*. Let E denote the set of all unit (row) vectors of size *L*, i.e.,

(8) $$E=\left\{e\in \mathrm{GF}{\left(2\right)}^{L}|\mathrm{wt}\left(e\right)=1\right\},$$

where wt(e) represents the Hamming weight of vector e. We aim at guessing out those error symbols belonging to E.

In the design of vector RLNC, for every coded packet $m_{P+d}=\sum_{j=1}^{P}K_{d,j}\circ m_{j}$, d≥1, we need to impose the following conditions on the random coding coefficient matrices.

(∗) All rows in $K_{d,j}$, 1≤j≤P, are distinct nonzero vectors, and none of them is a unit vector; that is,
  (9) $$|{⋃}_{u\in K_{d,j},1\leq j\leq P}\left\{u\right\}|=PL,\left\{{⋃}_{u\in K_{d,j},1\leq j\leq P}\left\{u\right\}\cap E\right\}=\phi ,$$
  where $u\in K_{d,j}$ traverses all the rows in the coding coefficient matrix $K_{d,j}$.

Since the global encoding kernel $F_{P+d}$ for coded packet $m_{P+d}$ is $F_{P+d}={\left[K_{d,1}^{T}\;K_{d,2}^{T}\;\ldots \;K_{d,P}^{T}\right]}^{T}$, condition (∗) equivalently states that all row vectors in $F_{P+d}$ are distinct, and not equal to any unit vector. The primary motivation for choosing the coding coefficient matrices subject to (∗) is that all ${\mathrm{eK}}_{d,j}$, 1≤j≤P and e∈E, constitute PL distinct row vectors. As a consequence, compared with estimating error vectors ${\tilde{e}}_{j}$, j∈J through the general formulation in (5)–(7), the syndrome $s_{d}$ obtained from received coded packet ${\hat{m}}_{P+d}=\sum_{j=1}^{P}K_{d,j}\circ m_{j}+e_{P+d}$ subject to (∗) offers a far more efficient alternative, as elaborated below.

**Efficient estimate of ${\tilde{e}}_{j}$**. Consider a vector RLNC scheme over $\mathrm{GF}{\left(2\right)}^{L}$ subject to condition (∗), equipped with the GRAND-assisted decoding routine formulated in the previous section. Upon receiving a new packet ${\hat{m}}_{P+d}$, instead of solving (5)–(7), receiver *r* performs the following concise procedure to obtain ${\tilde{e}}_{j}=\left[{\tilde{e}}_{j,1},\ldots ,{\tilde{e}}_{j,S}\right]$, j∈J, based on the syndrome $s_{d}=\left[s_{d,1},\ldots ,s_{d,S}\right]$:

- As initialization, set estimated error symbol ${\tilde{e}}_{j,i}=0$ for all j∈J and 1≤i≤S.
- For every nonzero symbol $s_{d,i}$ in $s_{d}$, 1≤i≤S, perform the next two steps.
- If $s_{d,i}$ is a unit vector, then set ${\tilde{e}}_{(P+d),i}=e$;
- Otherwise, check whether $s_{d,i}$ is identical to a row vector u in $K_{d,j}$, j∈J. If there exists such u, say in $K_{d,j^{\prime }}$ with $j^{\prime }\in J$, then set ${\tilde{e}}_{j,i}=e$.
- When the above two steps are performed for all nonzero $s_{d,i}$, 1≤i≤S, we obtain estimated error vectors ${\tilde{e}}_{j}$, j∈J.

The above process to obtain ${\tilde{e}}_{j}$ only involves the injective mapping from every $s_{d,i}$ to a vector in $K_{d,j}$, j∈J upon existence, so it is considered to take negligible computational overhead. It is worth noticing that ${\tilde{e}}_{j}$, j∈J obtained by the above process do not necessarily satisfy (5), but (6) and (7) always hold.

We next analyze the scenario where the efficiently estimated ${\tilde{e}}_{j}$, j∈J, from a syndrome $s_{d}$ is equal to $e_{j}$. As every syndrome $s_{d}$ is exploited independently in the efficient estimate, and according to Equation (3), syndrome $s_{d}$ does not contain any information of coded packets ${\hat{m}}_{P+1},\ldots ,{\hat{m}}_{P+d-1}$. Hence, only those $e_{j}$ with j∈{1,2,…,P}∪{P+d} are possible to correct (i.e., ${\tilde{e}}_{j}=e_{j}$) based on syndrome $s_{d}$. Notice that ${\tilde{e}}_{j}=e_{j}$ if and only if ${\tilde{e}}_{j,i}=e_{j,i}$ at every symbol location 1≤i≤S. For every 1≤i≤S with $e_{j,i}\neq 0$, ${\tilde{e}}_{j,i}=e_{j,i}$ holds if and only if

(10) $$e_{j,i}\in E,e_{j^{\prime },i}=0\;\forall j^{\prime }\in J\setminus \left\{j,P+1,\ldots ,P+d-1\right\}.$$

For every 1≤i≤S with $e_{j,i}=0$, we need ${\tilde{e}}_{j,i}=0$. Even though ${\tilde{e}}_{j,i}$ is set to 0 as initialization, it will be set to a nonzero vector e in E if $eK_{d,j}=s_{d,i}$. In this case, some nonzero error symbols at symbol location *i* are erroneously estimated to the 1-bit error symbol ${\tilde{e}}_{j,i}\in E$, so that ${\tilde{e}}_{j,i}\neq e_{j,i}=0$ and ${\tilde{e}}_{j}\neq e_{j}$. It turns out that ${\tilde{e}}_{j,i}=0$ whenever $e_{j,i}=0$ if and only if

(11) $$eK_{d,j}\neq s_{d,i}\;\forall e\in E,$$

where $K_{d,j}$ is assumed to be $I_{L}$ when j=P+d. Condition (11) is equivalent to stating that $s_{d,i}$ is not identical to any row vector of $K_{d,j}$. The above-characterized necessary and sufficient conditions for ${\tilde{e}}_{j}=e_{j}$ are summarized in the following proposition.

**Proposition 1.**  *Consider a vector RLNC scheme subject to condition (*∗*). When ${\tilde{e}}_{j}$, j∈J is obtained by the efficient estimate process based on $s_{d}$, a receiver is able to correct nonzero $e_{j}$; that is, ${\tilde{e}}_{j}=e_{j}$, if and only if the following holds.*

- *j∉{P+1,…,P+d−1}.*
- *For every symbol location 1≤i≤S with $e_{j,i}\neq 0$, condition (*10*) holds.*
- *For every symbol location 1≤i≤S with $e_{j,i}=0$, condition (*11*) holds.*

*Since condition (*10*) only involves error symbols and is not related to the design of coding coefficient matrices $K_{d,j}$, $\mathrm{Pr}\{{\tilde{e}}_{j,i}=e_{j,i}|e_{j,i}\neq 0\}$ keeps the same for all vector RLNC schemes subject to (*∗*).*

Based on Proposition 1, the probability $\mathrm{Pr}\{{\tilde{e}}_{j}=e_{j}\}$ for a receiver to correctly guess $e_{j}$ can be explicitly characterized, as long as the underlying channel model that determines the distribution of $e_{j}$ is given. In particular, when the memoryless BSC is considered, the sparse error pattern represented by Equation (10) occurs with high probability, so that $\mathrm{Pr}\{{\tilde{e}}_{j}=e_{j}\}$ is also high. As we do not focus on any particular channel model in the design of efficient estimate of ${\tilde{e}}_{j}$, the theoretical analysis of $\mathrm{Pr}\{{\tilde{e}}_{j}=e_{j}\}$ is beyond the scope of this paper.

To support the efficient estimation of ${\tilde{e}}_{j}$, condition (∗) must be satisfied by the coding coefficient matrices in vector RLNC. Compared with the scalar RLNC scheme with efficient GRAND-assisted decoding proposed in [26], condition (∗), formulated within the more general vector RLNC framework, provides greater design flexibility. Different choices of $K_{d,j}$ subject to (∗) may result in different coding complexities and distinct linear independence structures among global encoding kernels, thereby leading to different completion delay performances. Therefore, the design of vector RLNC schemes subject to (∗) warrants further investigation. In the following two subsections, we present two design instances with different features.

### 4.2. The First Design Instance of Vector RLNC Scheme Subject to (*∗*)

In this subsection, based on the matrix representation of finite field GF($2^{L}$) (see, e.g., [23,30]), we introduce the first explicit design instance of vector RLNC subject to condition (∗).

Let $p\left(x\right)=x^{L}+a_{L-1}x^{L-1}+\ldots +a_{1}x+1$ be a primitive polynomial of degree *L* over GF(2), and $\alpha \in \mathrm{GF}(2^{L})$ be a root of p(x). Consequently, $\alpha^{1},\alpha^{2},\ldots ,\alpha^{2^{L}-1}\left(=1\right)$ constitute $2^{L}-1$ distinct nonzero elements in GF($2^{L}$). Denote by A the following L×L companion matrix over GF(2) of p(x):

(12) $$A=\left[\begin{array}{cc} p & 1\\ I_{L-1} & 0 \end{array}\right],$$

where $p=[a_{L-1},a_{L-2},\ldots ,a_{1}]$. Then, the set

(13) $$M=\{0,A,A^{2},\ldots ,A^{2^{L}-2}\left(=I_{L}\right)\}$$

forms a matrix representation of GF($2^{L}$), where A plays the same role as α. Specifically, if $A(\alpha^{j})$ represents the matrix representation of $\alpha^{j}$ with A(α)=A, we have

(14) $$A\left(\alpha^{j}\right)=A^{j},\;\forall j\geq 0.$$

Define the following set K of L×L matrices

(15) $$K=\{A^{L},A^{2L},\ldots ,A^{(\left\lfloor \frac{2^{L}-1}{L}\right\rfloor -1)L}\}.$$

Let $C_{1}$ denote the vector RLNC scheme with $K_{d,1},\ldots ,K_{d,P}$ randomly and independently selected from K for each d≥1. For every coded packet, it takes $P(L-\left\lfloor \log_{2}L\right\rfloor )$ bits to store the information of *P* coding coefficient matrices.

**Proposition 2.**  *Condition (*∗*) holds for the vector RLNC scheme $C_{1}$.*

**Proof.**  For $0\leq j\leq 2^{L}-2$, denote by $p_{j}=\left[a_{j,L-1},a_{j,L-2},\ldots ,a_{j,0}\right]$ the (unique) polynomial representation of $\alpha^{j}$; that is, $\alpha^{j}=\sum_{l=0}^{L-1}a_{j,l}\alpha^{l}$. In this way, all $p_{j}$, $0\leq j\leq 2^{L}-2$, constitute the $2^{L}-1$ distinct nonzero vectors in $\mathrm{GF}{\left(2\right)}^{L}$. It is known (see, e.g., [23]) that

(16) $$A^{j}={\left[p_{j}^{T},p_{j+1}^{T},\ldots ,p_{j+L-1}^{T}\right]}^{T},$$

where $p_{j+l}$ represents $p_{j+l+1-2^{L}}$ whenever $j+L\geq 2^{L}-1$. This implies that all rows in all matrices given by (∗) are distinct. Since $p_{0},p_{1},\ldots ,p_{L-1}$ are the *L* unit vectors in $\mathrm{GF}{\left(2\right)}^{L}$, (16) further implies that only $A^{j}$, 0≤j≤L−1 and $2^{L}-L-1\leq j\leq 2^{L}-2$ contain rows equal to unit vectors. As these $A^{j}$ do not belong to K, there is not any row in any matrix in K equal to a unit vector. We can now conclude that for each d≥1, the *P* random coding coefficient matrices $K_{d,1},\ldots ,K_{d,P}$ satisfy condition (∗). □

Based on Proposition 2, as long as the code dimension *L* is set to satisfy $\lfloor \frac{2^{L}-1}{L}\rfloor >P$, the efficient process to estimate ${\tilde{e}}_{j}$ applies to the vector RLNC scheme $C_{1}$. Under the same *L*, let $C_{1}^{\prime }$ denote the scalar RLNC scheme over GF($2^{L}$) with the coding coefficients $k_{d,1},\ldots ,k_{d,P}\in \mathrm{GF}\left(2^{L}\right)$ for every coded packet $m_{P+d}=\sum_{j=1}^{P}k_{d,j}m_{j}$ randomly and distinctively from the following set

(17) $$K^{\prime }=\left\{\alpha^{L},\alpha^{2L},\ldots ,\alpha^{(\left\lfloor \frac{2^{L}-1}{L}\right\rfloor -1)L}\right\}.$$

As every $A^{j}=A\left(\alpha^{j}\right)$ in K is the matrix representation of $\alpha^{j}$ in $K^{\prime }$, a d×P matrix ${\left[k_{i,j}\right]}_{1\leq i\leq d,1\leq j\leq P}$ over GF($2^{L}$) with $k_{i,j}\in K^{\prime }$ is full rank if and only if the dL×PL matrix ${\left[A\left(k_{i,j}\right)\right]}_{1\leq i\leq d,1\leq j\leq P}$ with $A(k_{i,j})\in K$ over GF(2) is full rank. It turns out that both the vector RLNC scheme $C_{1}$ and the scalar RLNC scheme $C_{1}^{\prime }$ have the same distribution of completion delay.

The scalar RLNC scheme $C_{1}^{\prime }$ over GF($2^{L}$) can be equipped with the efficient GRAND-assisted decoding procedure proposed in [26], so that estimated error vectors, denoted by ${\tilde{e}}_{j}^{\prime }$, j∈J can be guessed with negligible computational overhead from syndromes. Even though the necessary and sufficient conditions for ${\tilde{e}}_{j}^{\prime }$ equal to $e_{j}$ were not explicitly given in [26], we remark here that the conditions in Proposition 1 apply to ${\tilde{e}}_{j}^{\prime }$ as well. Consequently, we have the following proposition.

**Proposition 3.**  *For the vector RLNC scheme $C_{1}$ and the counterpart scalar RLNC scheme $C_{1}^{\prime }$, they have the same distribution of completion delay. When $C_{1}$ and $C_{1}^{\prime }$ are equipped with the efficient GRAND-assisted decoding procedure respectively proposed in the previous subsection and in *[26]*, $C_{1}$ and $C_{1}^{\prime }$ also have the same distribution of completion delay, regardless of the channel modeling for bit-level transmission.*

Although the completion delay performance of the efficient GRAND-assisted vector RLNC scheme $C_{1}$ is identical to that of the counterpart scalar RLNC scheme $C_{1}^{\prime }$, our design yields a clear advantage in coding computational complexity. It has been theoretically analyzed in [23] that the theoretical coding complexity of vector RLNC over $\mathrm{GF}{\left(2\right)}^{L}$ with nonzero coding coefficient matrices randomly selected from M in (13) can be halved compared with scalar RLNC with nonzero coding coefficients randomly selected from $\mathrm{GF}(2^{L})$.

For brevity, assume every packet consists of 1 symbol; that is, it consists of *L*-bits. Given a binary row vector $m_{j}=\left[m_{j,L-1},\ldots ,m_{j,0}\right]$ of length *L*, let $m_{j}=\sum_{l=0}^{L-1}m_{j,l}\alpha^{l}$ denote the corresponding element in $\mathrm{GF}(2^{L})$, where α denotes the same primitive element as in (17). By adopting a similar argument to [23], we theoretically compare the average number of exclusive OR (XOR) operations involved in generating a vector RLNC packet $m_{P+d}=\sum_{j=1}^{P}m_{j}K_{d,j}$ and in generating a scalar RLNC packet $m_{P+d}=\sum_{j=1}^{P}m_{j}k_{d,j}$, with $K_{d,1},\ldots ,K_{d,P}$ randomly and distinctively selected from K in (15), and $k_{d,1},\ldots ,k_{d,P}$ randomly and distinctively selected from $K^{\prime }$ in (17). Same as in the proof of Proposition 2, for element $\alpha^{i}\in \mathrm{GF}\left(2^{L}\right)$, $0\leq i\leq 2^{L}-2$, denote by $p_{i}=\left[a_{i,L-1},a_{i,L-2},\ldots ,a_{i,0}\right]$ its unique polynomial representation; that is, $\alpha^{i}=\sum_{l=0}^{L-1}a_{i,l}\alpha^{l}$.

For the vector RLNC scheme $C_{1}$ to generate $m_{P+d}=\sum_{j=1}^{P}m_{j}K_{d,j}$, it takes $N_{1}-L$ XORs, where $N_{1}$ denotes the total number of nonzero entries in the corresponding global encoding kernel $F_{P+d}={\left[K_{d,1}^{T}\;\ldots \;K_{d,P}^{T}\right]}^{T}$. Since the *P* coding coefficient matrices $K_{d,1},\ldots ,K_{d,P}$ satisfy condition (∗), based on the characterization of $A^{j}$ in (16), it can be deduced that the expected value of $N_{1}$ is

(18) $$\begin{array}{cc} E[N_{1}]= & \frac{P}{\lfloor \frac{2^{L}-1}{L}\rfloor -1}\sum_{i=L}^{\lfloor \frac{2^{L}-1}{L}\rfloor L-1}\mathrm{wt}\left(p_{i}\right)\\ = & \frac{P}{\lfloor \frac{2^{L}-1}{L}\rfloor -1}\left(L2^{L-1}-L-\sum_{i=\lfloor \frac{2^{L}-1}{L}\rfloor L}^{2^{L}-2}\mathrm{wt}\left(p_{i}\right)\right) \end{array}$$

We now consider the case of scalar coding over GF($2^{L}$). As α is a root of the defined primitive polynomial $p\left(x\right)=x^{L}+a_{L-1}x^{L-1}+\ldots a_{1}x+1$,

(19) $$\alpha^{L}=1+\sum_{l=1}^{L-1}a_{l}\alpha^{l}.$$

Thus, for i≥0, it takes iη XORs to compute $m_{j}\alpha^{i}$, where η denotes the number of nonzero coefficients in $\left\{a_{1},\ldots ,a_{L-1}\right\}$. Moreover, notice that in computing $m(\alpha^{i}+\alpha^{i+i^{\prime }})$ with $i,i^{\prime }>0$, it is equivalent to compute $m\alpha^{i}+\left(m\alpha^{i}\right)\alpha^{i^{\prime }}$, so it takes $(i+i^{\prime })\eta +L$ XORs. Consequently, for a coefficient $\alpha^{i}=\sum_{l=0}^{L-1}a_{i,l}\alpha^{l}\in \mathrm{GF}\left(2^{L}\right)$ with the unique polynomial representation $p_{i}=\left[a_{i,L-1},a_{i,L-2},\ldots ,a_{i,0}\right]$, it takes $l_{i,\max}\eta +\left(\mathrm{wt}\left(p_{i}\right)-1\right)L$ XORs to compute $m_{j}\alpha^{i}$, where $l_{i,\max}$ represents the largest degree in $\alpha^{i}=\sum_{l=0}^{L-1}a_{i,l}\alpha^{l}$ with a nonzero coefficient; that is,

(20) $$l_{i,\max}=\max_{a_{i,l^{\prime }}\neq 0}l^{\prime }.$$

Let $N_{1}^{\prime }$ denote the total number of XORs involved in generating $m_{P+d}=\sum_{j=1}^{P}m_{j}k_{d,j}$ based on the previously analyzed arithmetic. When $k_{d,1},\ldots ,k_{d,P}$ are randomly and distinctively selected from $K^{\prime }$ in (17),

(21) $$E\left[N_{1}^{\prime }\right]=\left(P-1\right)L+\frac{P}{\lfloor \frac{2^{L}-1}{L}\rfloor -1}\sum_{i=1}^{\lfloor \frac{2^{L}-1}{L}\rfloor -1}\left(l_{iL,\max}\eta +\left(\mathrm{wt}\left(p_{iL}\right)-1\right)L\right).$$

As an explicit comparison based on (18) and (21), for the case P=10, L=8 and $\alpha^{8}+\alpha^{4}+\alpha^{3}+\alpha^{2}+1=0$, it takes 512 XORs on average to generate $m_{P+d}=\sum_{j=1}^{P}m_{j}k_{d,j}$, with $k_{d,1},\ldots ,k_{d,P}$ randomly and distinctively selected from $K^{\prime }$ in (17). It takes 321 XORs on average to generate $m_{P+d}=\sum_{j=1}^{P}m_{j}K_{d,j}$, with $K_{d,1},\ldots ,K_{d,P}$ satisfying (∗), which yields a 37.3% reduction in coding computational complexity.

### 4.3. The Second Design Instance of Vector RLNC Scheme Subject to (∗)

In addition to the one presented in the previous subsection, we next present another design instance of vector RLNC subject to condition (∗). Let 2<w≤L/2. Define V as the set of all *L*-dimensional binary row vectors of Hamming weight $w^{\prime }$, $2\leq w^{\prime }\leq w$; that is,

(22) $$\begin{array}{c} V=\left\{u\in \mathrm{GF}{\left(2\right)}^{L}\;|\;2\leq \mathrm{wt}\left(u\right)\leq w\right\}. \end{array}$$

Consider the vector RLNC scheme $C_{2}$ with the rows in $K_{d,1},\ldots ,K_{d,P}$, for each d≥1, randomly and distinctively selected from V. To ensure the existence of such $C_{2}$, the number of vectors in V must satisfy

(23) |V|=∑i=2wLi≥PL.

Accordingly, for every coded packet, it takes PL⌈log2(∑i=2wLi)⌉ bits to store the information of *P* coding coefficient matrices.

**Proposition 4.**  *Condition (∗) naturally holds for the vector RLNC scheme $C_{2}$.*

For an erroneously received packet $m_{j}$, j∈J, Proposition 1 in Section 4.1 characterizes the necessary and sufficient conditions for the efficiently estimated error vector ${\tilde{e}}_{j}$ equal to $e_{j}$. Based on this, we remark that the probability $\mathrm{Pr}\{{\tilde{e}}_{j}=e_{j}\}$ for $C_{2}$ is slightly different from that for the vector RLNC scheme $C_{1}$ presented in the previous subsection, because different selections of $K_{d,e}$ lead to different (yet small) probabilities of Equation (11) occurring. As an illustration, assume that the bit-level transmission follows the BSC, the packet erasure probability is $1-p_{r}=0.2$, and for both schemes, there are P=10 original packets, the code dimension is L=8, and the packet length is M=1024. Numerical results show that upon receiving the first coded packet $m_{P+1}$ and based on the first syndrome $s_{1}$, $\mathrm{Pr}\{{\tilde{e}}_{j}=e_{j}\}$ is approximately 0.988 for both $C_{1}$ and $C_{2}$. When the packet erasure probability $1-p_{r}$ is changed to 0.64 and the packet length is changed to M=256 (which means the bit error rate significantly increases), $\mathrm{Pr}\{{\tilde{e}}_{j}=e_{j}\}=0.588$ for $C_{1}$ and $\mathrm{Pr}\{{\tilde{e}}_{j}=e_{j}\}=0.580$ for $C_{2}$.

One feature of the vector RLNC scheme $C_{2}$ is that its coding coefficient matrices are sparser than the ones in $C_{1}$, so that lower coding complexity than $C_{1}$ can be achieved. We now theoretically analyze the number of XORs involved in generating a coded packet $m_{P+d}=\sum_{j=1}^{P}m_{j}K_{d,j}$ for $C_{2}$. Denote by $N_{2}$ the total number of nonzero entries in the global encoding kernel $F_{d}={\left[K_{d,1}^{T}\;\ldots \;K_{d,P}^{T}\right]}^{T}$. The average number of XORs involved in generating $m_{P+d}=\sum_{j=1}^{P}m_{j}K_{d,j}$ is $E[N_{2}]-L$, with

(24) E[N2]=P∑l=2wLl∑l=2wLll.

As an explicit comparison, for the case P=10, L=8, and w=3, $C_{2}$ takes 213.3 XORs on average to generate $m_{P+j}=\sum_{j=1}^{P}m_{j}K_{d,j}$, a further 33.6% reduction in computational complexity compared with the vector RLNC scheme $C_{1}$ presented in the previous subsection.

As a cost for the sparsity of the coding coefficient matrices, the scheme $C_{2}$ exhibits weaker linear independence properties among coded packets than $C_{1}$; that is, for *d* coded packets $m_{P+1},\ldots ,m_{P+d}$, the full-rank probability; that is, $\mathrm{Pr}\{\mathrm{rank}\left({\left[F_{P+j}\right]}_{1\leq j\leq d}\right)=\min\left\{PL,Pd\right\}\}$, of $C_{2}$ is smaller than that of $C_{1}$. For instance, in the setting P=10 and L=8, for $C_{1}$, the full-rank probability for *P* coded packets is as high as

(25) $$\mathrm{Pr}\{\mathrm{rank}\left({\left[F_{P+j}\right]}_{1\leq j\leq P}\right)=PL\}=0.996,$$

while for $C_{2}$ with w=3, the full-rank probability for *P* coded packets decreases to

(26) $$\mathrm{Pr}\{\mathrm{rank}\left({\left[F_{P+j}\right]}_{1\leq j\leq P}\right)=PL\}=0.285.$$

For $C_{2}$ with w=3, it requires P+1 coded packets to achieve the full-rank with high probability; that is,

(27) $$\mathrm{Pr}\{\mathrm{rank}\left({\left[F_{P+j}\right]}_{1\leq j\leq P+1}\right)=PL\}=0.996.$$

The weaker linear independence among coded packets in $C_{2}$ increases the average completion delay at a receiver. To explicate this, assume receiver *r* has obtained a set I of *P* error-free packets for the first time, which consists of a subset $I_{1}$ of original packets and a subset $I_{2}$ of coded packets. Let Δ denote the difference in completion delay at receiver *r* between schemes $C_{2}$ and $C_{1}$. Under the simplified assumptions that (i) for both schemes, it takes *E* transmissions on average for receiver *r* to obtain one error-free coded packet; (ii) for all P≥1, $\mathrm{Pr}\{\mathrm{rank}\left({\left[F_{P+j}\right]}_{1\leq j\leq P}\right)=PL\}\approx 1$ for $C_{1}$, $\mathrm{Pr}\{\mathrm{rank}\left({\left[F_{P+j}\right]}_{1\leq j\leq P+1}\right)=PL\}\approx 1$ for $C_{2}$, and $\mathrm{Pr}\{\mathrm{rank}\left({\left[F_{P+j}\right]}_{1\leq j\leq P}\right)=PL\}$ keeps the same for $C_{2}$, we have

(28) $$E[\Delta ||I_{1}\left|\neq P\right]=\left(1-\mathrm{Pr}\left\{\mathrm{rank}\left({\left[F_{P+j}\right]}_{1\leq j\leq P}\right)=PL\right\}\right)E.$$

The above equation implies that due to the smaller full-rank probability among coded packets in $C_{2}$, when $I_{1}$ does not contain all *P* original packets, with probability $1-\mathrm{Pr}\{\mathrm{rank}\left({\left[F_{P+j}\right]}_{1\leq j\leq P}\right)=PL\}$, receiver *r* has to collect one more error-free coded packet (that is, $P-|I_{1}|+1$ coded packets in total) for RLNC decoding in $C_{2}$, which takes *E* more transmissions on average. Since $E[\Delta ||I_{1}|=P]=0$, we further have

(29) $$E\left[\Delta \right]=\mathrm{Pr}\{|I_{1}\left|\neq P\right\}\left(1-\mathrm{Pr}\left\{\mathrm{rank}\left({\left[F_{P+j}\right]}_{1\leq j\leq P}\right)=PL\right\}\right)E.$$

Without the assistance of the GRAND process, $\mathrm{Pr}\{|I_{1}\left|\neq P\right\}=1-p_{r}^{P}$ and $E=1/p_{r}$. In comparison, with the efficient GRAND process, upon receiving a coded packet, receiver *r* has an extra (high) chance of directly obtaining all *P* error-free original packets or obtaining more error-free coded packets, so that $\mathrm{Pr}\{|I_{1}|\neq P\}$ increases and *E* decreases. It can be shown that compared with the classical scenario of vector RLNC without the assistance of GRAND, the effect of weaker linear independence among coded packets on the completion delay performance for $C_{2}$ is reduced when the GRAND process is taken into account. In other words, the expected completion delay difference E[Δ] decreases when the vector RLNC schemes are equipped with the efficient GRAND process. We shall numerically demonstrate this in the next section.

## 5. Numerical Analyses

When the channel model for bit-level transmission is assumed to be the memoryless BSC, extensive theoretical and numerical analyses have been conducted in [26] for the completion delay performance of the efficient GRAND-assisted scalar RLNC scheme $C_{1}^{\prime }$ over GF($2^{L}$) presented in Section 4.2. According to Proposition 3, these analyses also hold for the vector RLNC scheme $C_{1}$ equipped with the efficient GRAND process proposed in the Section 4.2.

In this section, under the same assumption of the BSC channel for bit-level transmission, we shall numerically compare the vector RLNC schemes $C_{1}$ and $C_{2}$ satisfying condition (∗) constructed in the previous section. All of the curves shown in the figures are simulation-based. Each data point is averaged over $10^{6}$ independent Monte Carlo trials. In the figure legend, the label “Scheme 1” corresponds to the first scheme $C_{1}$ with the setting P=10 and L=8, and “Scheme 2, w=3” represents the second scheme $C_{2}$ with the setting P=10, L=8 and w=3. The legend “w/GRAND” denotes the considered RLNC scheme equipped with the efficient GRAND process to estimate error vectors, while “w/o GRAND” refers to the considered RLNC scheme without GRAND-assistance; that is, a received packet ${\hat{m}}_{j}$ will be discarded if it is not equal to $m_{j}$.

In Section 4.3, we have numerically validated that for j∈J, there is only slight difference for $\mathrm{Pr}\{{\tilde{e}}_{j}=e_{j}\}$ between $C_{1}$ and $C_{2}$. Next, we numerically compare the distribution for $N_{r}$, which represents the number of packets (out of P+1 packets) that cannot be correctly recovered by the efficient GRAND process upon receiving the first coded packet $m_{P+1}$ at receiver *r*. In particular,

(30) $$\mathrm{Pr}\left\{N_{r}=0\right\}=\mathrm{Pr}\left\{{⋃}_{j\in J}{\tilde{e}}_{j}=e_{j}\right\}.$$

The distribution of $N_{r}$ is closely related to the completion delay performance.

**Figure 1:**
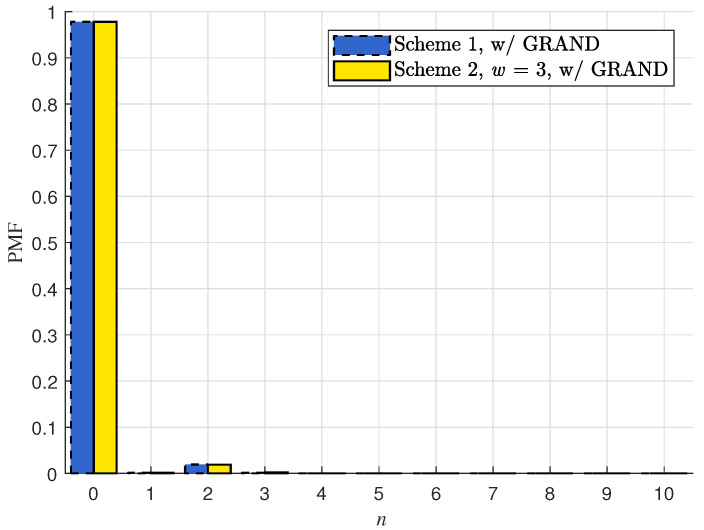
The PMF of $N_{r}$ for the two vector RLNC schemes subject to (∗) proposed in the previous section, under the setting P=10, L=8, $p_{r}=0.8$ and M=1024.

Figure 1 depicts the probability mass function (PMF) of $N_{r}$ for $C_{1}$ and for $C_{2}$ under the assumption that the packet length is M=1024 and the packet erasure probability is $1-p_{r}=0.2$ (corresponding to the bit error rate of $1-p_{r}^{1/M}=2.1789\times 10^{-4}$). The results show that the PMFs $\mathrm{Pr}\{N_{r}=n\}$ of the two schemes are nearly identical, and $\mathrm{Pr}\{N_{r}=0\}=0.9779$, implying that the receiver can recover all *P* original packets with high probability after the efficient GRAND process based on the first syndrome $s_{1}$.

**Figure 2:**
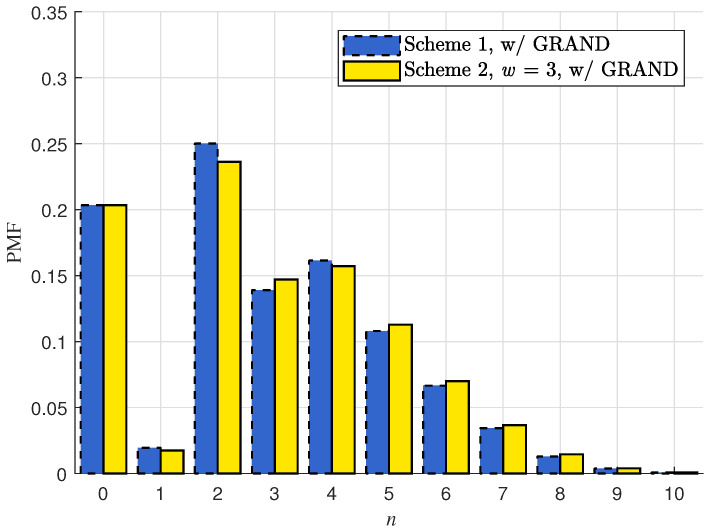
The PMF of $N_{r}$ for the two vector RLNC schemes subject to (∗) proposed in the previous section, under the setting P=10, L=8, $p_{r}=0.36$ and M=256.

Figure 2 compares the PMF between the two schemes with shorter packet length M=256 and under the less reliable channel condition $p_{r}=0.36$ (corresponding to the bit error rate of $1-p_{r}^{1/M}=3.98\times 10^{-3}$). As the bit error rate increases by over an order of magnitude in the new setting, the probability of $N_{r}=0$ dramatically decreases to 0.21. Moreover, a slight difference can be observed for the distribution of $N_{r}$ between the two schemes. Similar to the analysis about the slight difference of $\mathrm{Pr}\{{\tilde{e}}_{j}=e_{j}\}$ between the two schemes in Section 4.3, the slight difference in the distribution of $N_{r}$ between the two schemes is also because different selection of coding coefficient matrices $K_{d,j}$ leads to different yet small probabilities of (11) occurring.

**Figure 3:**
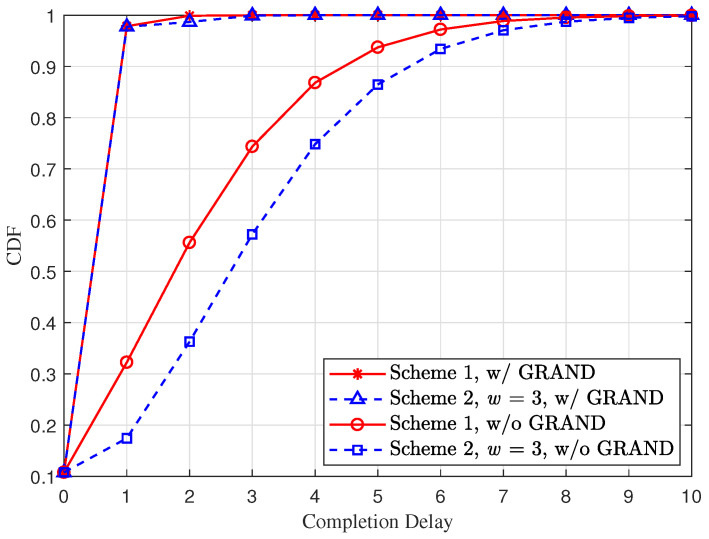
The CDF of the completion delay at receiver *r* for different vector RLNC schemes subject to (∗) under the setting P=10, L=8, $p_{r}=0.8$ and M=1024.

Next, we numerically compare the cumulative distribution function (CDF) of the completion delay at receiver *r* for the two schemes. Figure 3 depicts the CDF of completion delay under the setting of packet length M=1024 and packet erasure probability $1-p_{r}=0.2$, corresponding to a bit error rate of $1-p_{r}^{1/M}=2.179\times 10^{-4}$. We observe that the efficient GRAND process significantly enhances the completion delay performance for both vector RLNC schemes. In particular, upon receiving one coded packet, the efficient GRAND process significantly increases the successful recovery probability to 0.98 for both schemes. In addition, as illustrated in Figure 3, without the efficient GRAND process, there is a noticeable difference in completion delay between the two schemes. As analyzed in Section 4.3, this is attributed to the weaker linear independence and thus the lower full-rank probability among coded packets in Scheme $C_{2}$, which affects RLNC decoding. However, when the efficient GRAND process is taken into account, the performance gap between the two schemes diminishes. This is mainly because upon receiving the first coded packet, the receiver can directly obtain all *P* error-free original packets via the efficient GRAND process with high probability, so that the impact of different full-rank probabilities among coded packets between the two schemes becomes negligible.

**Figure 4:**
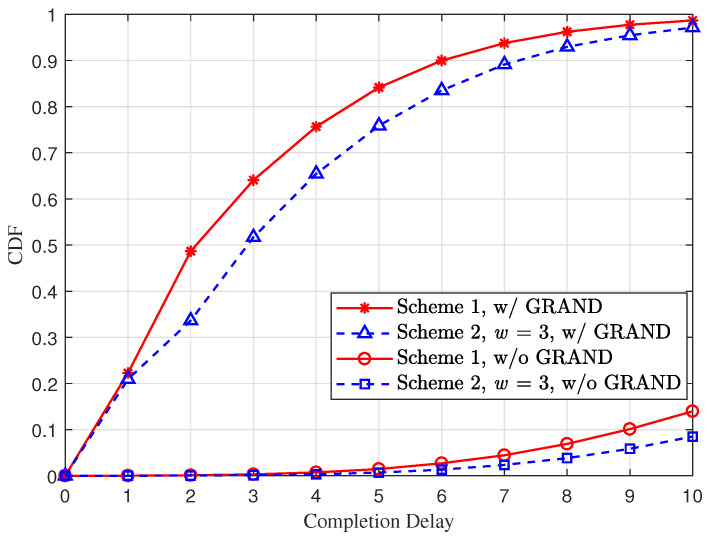
The CDF of the completion delay at receiver *r* for different vector RLNC schemes subject to (∗) under the setting P=10, L=8, R=1, $p_{r}=0.36$ and M=256.

Figure 4 depicts the CDF of completion delay at receiver *r* under the assumption that the packet length is M=256 and the packet erasure probability is $1-p_{r}=0.64$ (corresponding to the bit error rate of $1-p_{r}^{1/M}=3.98\times 10^{-3}$). In this scenario, a remarkable gain in the completion delay performance resulting from the efficient GRAND process can still be observed for both schemes. In contrast to the observations in Figure 3, the performance gap between the two schemes equipped with the efficient GRAND process becomes noticeable in Figure 4. This is because, compared with the setting in Figure 3, the current scenario has a much higher bit error rate, which reduces the probability of all *P* original packets being recovered via the efficient GRAND process. As a result, the weaker linear independence among coded packets in Scheme $C_{2}$ starts to affect the completion delay performance.

Table 1 compares the average completion delay at receiver *r* under three different settings. In all the settings, GRAND-assisted decoding significantly reduces the average completion delay for both schemes. Specifically, in Setting 1, the average completion delay is reduced by 63.5% for $C_{1}$ and by 71.7% for $C_{2}$, while in Setting 2, where *P* and *L* becomes larger and $p_{r}$ stays the same, the corresponding reductions are 74% and 77.8%. In Setting 3, where the packet erasure probability $1-p_{r}$ becomes much larger, the corresponding reductions further increase to 81.4% and 79.8%, respectively. Recall that in Section 4.3, the expected difference E[Δ] between schemes $C_{2}$ and $C_{1}$ is characterized in (29) under simplified assumptions. Without the efficient GRAND process, we have $\mathrm{Pr}\{|I_{1}\left|\neq P\right\}=1-p_{r}^{P}$ and $E=1/p_{r}$. Substituting these into (29) yields E[Δ]≈0.798 in Setting 1, E[Δ]≈0.869 in Setting 2, and E[Δ]≈1.986 in Setting 3, all of which are consistent with the numerical results in Table 1. With the efficient GRAND process, in Setting 1 and Setting 2, $\mathrm{Pr}\{|I_{1}|\neq P\}$ becomes close to zero, leading to a small E[Δ]. In Setting 3, $\mathrm{Pr}\{|I_{1}|\neq P\}$ is also significantly reduced compared with $1-0.36^{10}$, and *E* is smaller than $1/p_{r}$, which jointly decrease E[Δ]. These analytical observations and analyses are in line with the numerical results.

Lastly, based on the above observations, one may conclude that when the packet erasure probability and the corresponding bit error rate are high, Scheme $C_{1}$ achieves a better completion delay performance than Scheme $C_{2}$. This highlights a tradeoff between computational complexity and decoding performance in our design of vector RLNC schemes subject to condition (∗) in the last section. The first design instance, based on the matrix representation of GF$\left(2^{L}\right)$, inherits the properties of strong linear independence and high full-rank probability among coded packets in scalar RLNC over GF($2^{L}$). The second design instance, on the other hand, employs sparse coding coefficient matrices and significantly reduces coding complexity, at the cost of slightly degraded performance caused by the weaker linear independence and thus the lower full-rank probability among coded packets under poor channel conditions. For example, as shown in Table 1, under Setting 1 where the packet erasure probability is 0.2, the average completion delay is 0.915 for Scheme $C_{1}$ with GRAND-assistance and 0.929 for Scheme $C_{2}$ with GRAND-assistance, which corresponds to a degradation of approximately 1.5%.

## 6. Concluding Remarks

Building upon the results presented in this paper, the proposed framework of efficient GRAND-assisted vector RLNC provides flexibility in the design of coding coefficient sets. The two design instances presented in this paper exhibit different characteristics with regard to computational complexity and completion delay performance. The first design instance, based on the matrix representation of GF$\left(2^{L}\right)$, inherits the strong linear independence properties of scalar RLNC, and achieves the same completion delay performance as the counterpart scalar RLNC scheme, with reduced decoding complexity. The second design instance employs sparse coding coefficient matrices to further reduce computational complexity, at the cost of degraded completion delay performance under poor channel conditions due to weaker linear independence among coded packets. This tradeoff highlights the importance of coding coefficient design in vector RLNC, and the two schemes are applicable to different system conditions. In particular, Scheme $C_{1}$ is suitable for high error-rate conditions, while Scheme $C_{2}$ is more suitable for resource-constrained systems at the cost of a tolerable degradation in completion delay.

The proposed framework enables the design of coding schemes that satisfy the design rule while balancing computational complexity and completion delay performance. Although the BSC model is adopted for analysis and simulations in this paper, the framework is general and can be extended to other channel models. In particular, for channels with correlated errors, such as burst error channels, new designs of coding coefficient subsets may be required. This generality also opens up interesting directions for future work, where new code designs can be developed to further outperform GRAND-assisted scalar RLNC in terms of completion delay.

## Figures and Tables

**Table 1:** Average completion delay at receiver *r* of different RLNC schemes under Setting 1: P=10, L=8, M=1024, $p_{r}=0.8$; Setting 2: P=16, L=10, M=1020, $p_{r}=0.8$; and Setting 3: P=10, L=8, M=256, $p_{r}=0.36$.

	Settings	1	2	3
Scheme				
$C_{1}$, w/GRAND		0.915	1.039	3.305
$C_{2}$, w=3, w/GRAND		0.929	1.081	3.989
$C_{1}$, w/o GRAND		2.510	3.998	17.793
$C_{2}$, w=3, w/o GRAND		3.286	4.870	19.768

## Data Availability

The theoretical analysis and key numerical results are fully presented in this paper. Raw simulation data and code are available from the corresponding author upon reasonable request.

## Abbreviations

List of Symbols	
A:	The companion matrix of a primitive polynomial defined in (12).
E:	The set of all unit (row) vectors of size *L*.
$e_{j}$:	The error vector in received packet ${\hat{m}}_{j}$.
${\tilde{e}}_{j}$:	The estimated error vector of $e_{j}$.
$F_{P+d}$:	The global encoding kernel of packet $m_{P+d}$.
J:	The index of erroneously received packets.
K:	The set of coding coefficient matrices for vector RLNC defined in (15).
$K^{\prime }$:	The set of coding coefficients for scalar RLNC defined in (17).
$k_{d,j}$:	Random coding coefficients for scalar RLNC.
$K_{d,j}$:	Random coding coefficient matrices for vector RLNC.
*L*:	The number of bits in a symbol of a packet.
*M*:	The number of bits in a packet.
$m_{j}$:	The transmitted packet, which is a row vector of M/L symbols.
${\hat{m}}_{j}$:	The packet received by receiver *r*.
*P*:	The number of original packets.
$p_{r}$:	The probability of successfully receiving a packet at receiver *r*.
$p_{rb}$:	The probability of a bit in a packet being corrupted at receiver *r*.
*R*:	The number of receivers.
*S*:	The number of symbols in a packet, equal to M/L.
$s_{d}$:	The syndrome defined in (3).
V:	The set of vectors with limited Hamming weight defined in (22).
